# Emergency laparoscopic ileocecal resection for a low-grade appendiceal mucinous neoplasm with impending rupture: A case report

**DOI:** 10.1016/j.ijscr.2021.02.022

**Published:** 2021-02-09

**Authors:** Yusuke Kitagawa, Shunsuke Hamasaki, Toshiko Harada, Noriyasu Tamura, Akira Katsuno, Naoyuki Umetani

**Affiliations:** Department of Digestive Surgery, Kawakita General Hospital, 1-7-3, Asagaya-kita, Suginami-ku, Tokyo, 166-0001, Japan

**Keywords:** Low-grade appendiceal mucinous neoplasm, Appendiceal cancer, Magnetic resonance imaging, Laparoscopic ileocecal resection, Appendectomy, Case report

## Abstract

**Introduction and importance:**

We report the case of a patient with a low-grade appendiceal mucinous neoplasm (LAMN) who underwent emergency laparoscopic ileocecal resection to avoid the metastatic spread of tumor cells due to an impending rupture.

**Case presentation:**

A 55-year-old woman presented to our hospital with pain in the right lower quadrant of the abdomen. Computed tomography revealed a markedly tense appendiceal mucinous tumor with surrounding inflammation, and laboratory test results showed elevated serum C-reactive protein (7.47 mg/dL), indicating impending rupture of the appendix. Magnetic resonance imaging revealed nodules inside the appendix, suggesting the possibility of appendiceal cancer. We performed emergency laparoscopic ileocecal resection with regional lymph node dissection. The tumor was pathologically diagnosed as a LAMN without rupture.

**Clinical discussion:**

LAMN is classified as a clinically malignant tumor because it can cause pseudomyxoma peritonei due to perforation or the presence of residual tissue. Although an appendectomy would be appropriate for LAMN if the tumor margin is secured, ileocecal resection with lymph node dissection is necessary when preoperative discrimination of appendiceal cancer is impossible.

**Conclusion:**

Further studies of preoperative imaging for appropriate differential diagnosis were necessary.

## Highlights

• Appendiceal mucinous neoplasms account for less than 1% of all cancers.
• LAMNs have an aggressive biological potential.
• Preoperative diagnosis of appendiceal mucinous neoplasms is difficult.
• Here, we used emergency MRI to identify nodules in the appendix before operation.

## 1 Introduction

Appendiceal mucinous neoplasms are rare tumors, accounting for less than 1% of all cancers. They include a heterogeneous group of diseases with varying malignant potential as reflected in their different classification systems [1]. Low-grade appendiceal mucinous neoplasms (LAMNs) are characterized by a benign morphologic appearance and aggressive biological potential, as outlined in the recent edition of the World Health Organization (WHO) classification [2,3]. It is known that a prior appendiceal perforation or residual tissue during surgery causes pseudomyxoma peritonei (PMP). Some cases are diagnosed for the first time because of appendicitis, and accurate preoperative diagnosis, including histological examination, is difficult. Some cases are identified during other abdominal surgeries, suggesting that preoperative diagnosis, even with an imaging examination, is difficult. Ileocecal resection or right hemicolectomy is recommended to secure the surgical margin [4]. However, it is reported that an appendectomy is adequate even in the case of a positive surgical margin [5]. In addition, because the incidence of nodal spread of well-differentiated localized appendiceal tumors is less than 2%, lymph node dissection is deemed unnecessary [1]. However, it is difficult to differentiate LAMN from appendiceal cancer with possible lymph node metastasis before surgery. Imaging plays an important role in the diagnosis and management of mucinous appendiceal neoplasms. It might be helpful in the identification of asymptomatic neoplasms and detection of local invasion, impending or actual rupture, and metastatic spread of these tumors [6]. Nevertheless, it is difficult to distinguish LAMN and appendiceal cancer using imaging. In our case, emergency magnetic resonance imaging (MRI) revealed nodules in the appendix. We made a provisional diagnosis of appendiceal cancer, and laparoscopic ileocecal resection was performed with lymph node dissection. In this paper, we also describe five other cases of LAMN diagnosed in our hospital and present a literature review. This work has been reported in line with the SCARE 2020 criteria [7].

## 2 Presentation of case

A 55-year-old woman complaining of abdominal pain was referred to our hospital for further examination. She had a history of asthma and was allergic to iodine contrast agents. On physical examination of her abdomen, signs of peritonitis were observed. Laboratory test results revealed a C-reactive protein (CRP) level of 7.47 mg/dL (normal value, ≤0.3 mg/dL) and a carcinoembryonic antigen (CEA) level of 11.49 ng/mL (normal value, ≤5.0 ng/mL). Other laboratory results were within normal limits, including the carbohydrate antigen 19−9 (CA19−9) and 125 (CA125).

**Fig. 1:**
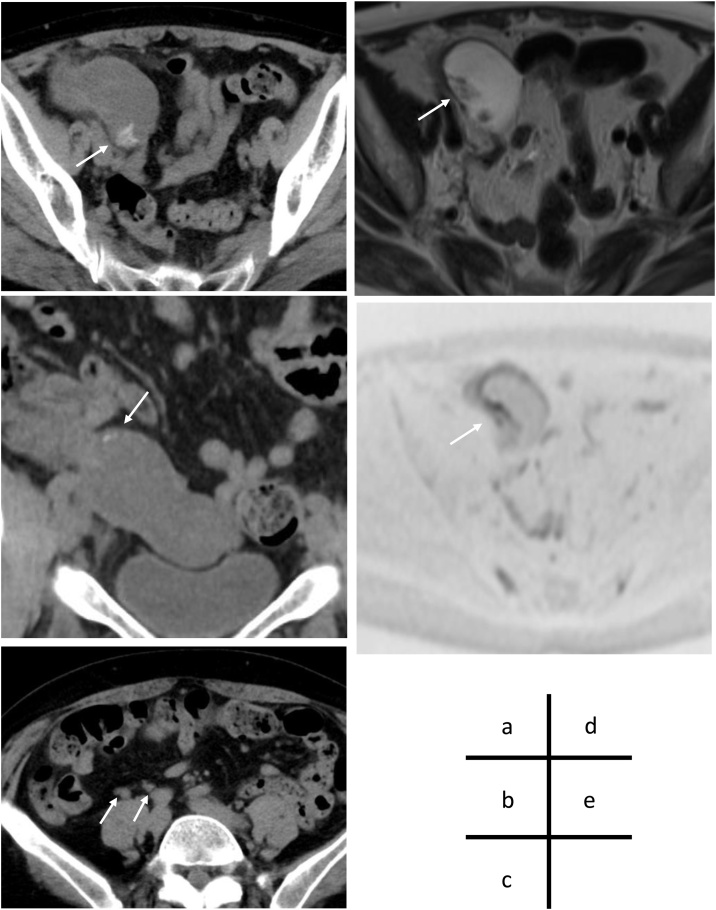
(a, b) Image of the appendix showing it was swollen up to 38 mm in diameter, and had a bright nodule inside (white arrow). (c) No ascites was observed; however, regional lymph nodes were enlarged (white arrow). (d, e) Magnetic resonance imaging (MRI) revealed a high-intensity nodular ridge on T2WI and accumulation on diffusion-weighted imaging (DWI, white arrow).

Computed tomography (CT) revealed a swollen mucin pool inside the appendix, which had a diameter of 38 mm. Appendiceal calcification was visualized. Although the surrounding adipose tissue was turbid, no evidence of PMP was found. Multiple ileocecal lymph nodes were enlarged (Fig. 1, a–c). Imminent perforation was suspected because the CRP was elevated, and the inflammation was spreading around the appendix. Furthermore, MRI revealed lobulated enlargement of the appendix, irregular internal wall thickening, and a mass-like structure. On diffusion-weighted imaging (DWI), a high-intensity signal was observed in a part of the appendiceal wall, indicating the possibility of an appendiceal mucinous adenocarcinoma (Fig. 1, d–e). Based on these findings, we decided to perform an emergency ileocecal resection with lymph node dissection.

**Fig. 2:**
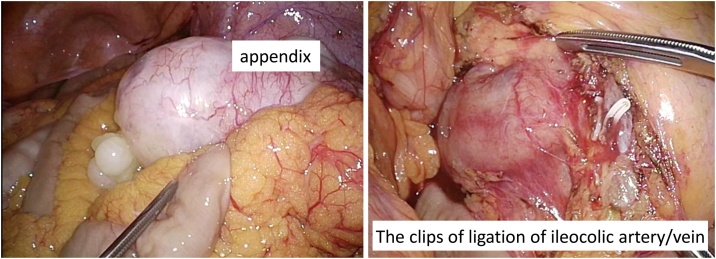
Although the appendix was swollen and tense, there was no ascites and no evidence of perforation. A jelly-like nodular protrusion was found at the tip of the appendix. Regional lymph node dissection was performed.

**Fig. 3:**
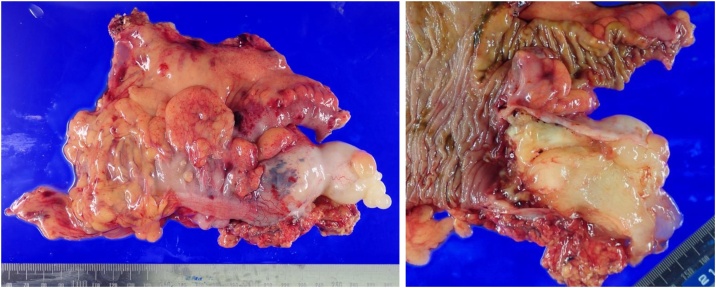
Image of the serosal surface that had turned white. There was no evidence of inflammation. The lumen of the appendix was filled with jelly-like contents.

**Fig. 4:**
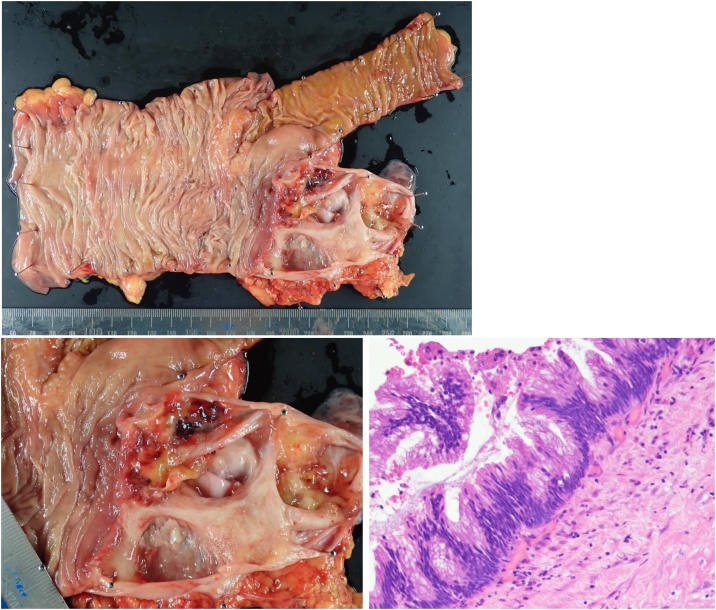
Macroscopically, the inside of the appendix had a septum with a soft nodule in part. Pathologically, there was a tumor gland duct rich in mucus production, but there was no evidence of atypical mucinous adenoma, destructive interstitial infiltration, or vascular invasion.

Intraoperative findings showed no ascites in the abdominal cavity and no suspicious nodules for PMP. There was a jelly-like nodule at the tip of the appendix. Ileocecal resection with regional lymph node dissection was performed laparoscopically (Fig. 2). Macroscopically, the appendiceal serosa was whitish, and mild inflammation was observed (Fig. 3). The appendiceal lumen was filled with a yellowish jelly and had a septum, and a soft nodule formation was observed. Pathologically, although tumor gland ducts rich in mucus production were observed, no mucinous carcinoma-like atypia, destructive interstitial invasion, or vascular invasion was observed. The mucin pool spread from the muscularis propria to the subserosa, and tumor ducts were found inside (Fig. 4). No lymph node metastasis was observed. Based on the above findings, we diagnosed LAMN. Postoperatively, the CEA level returned to normal.

## 3 Discussion

We present a case of LAMN in which emergency laparoscopic ileocecal resection with D3 lymph node dissection was performed. Preoperative CT and MRI suggested an epithelial tumor of the appendix.

LAMN confined to the appendiceal lumen do not show definitive malignant features. However, they can be malignant and proliferate outside the appendix and lead to PMP [8]. Although prompt diagnosis and subsequent surgery are required, preoperative diagnosis of appendiceal mucinous neoplasms is difficult.

There are varying reports regarding the treatment, depending on the pathological grade and extent of the lesion. However, it is not advisable to wait for colonoscopy to observe the orifice of the appendix. In clinical practice, cases in which the pathological grade is determined before the surgery are rare. We performed emergency laparoscopic ileocecal resection with lymph node dissection in our patient. In a study of 64 patients who underwent surgical treatment for LAMN, there was no significant difference in the results between appendectomy and colectomy, and the depth of invasion and progression of peritoneal metastasis were attributed to recurrence [4]. Among these, in four cases of appendectomy, the proximal margin was positive, and an additional resection was performed. All additional treatments were right hemicolectomy. Regarding the significance of the surgical margin, Arnason et al. reported 15 cases with positive margins after appendectomy for LAMN. Although nine patients were followed up carefully without additional treatment, no recurrence was observed, while additional resection was required in six cases [5]. However, considering that many cases with recurrence have PMP, for which the treatment is highly invasive, it is considered that additional treatment is desirable to achieve positive margins. Because the incidence of nodal spread of well-differentiated localized appendiceal tumor is less than 2%, most of the published surgical literature suggests that a simple appendectomy is sufficient for tumors exhibiting only local disease [1]. However, to clear the tumor margin, as in our case, ileocecal resection should be considered in case the tumor spreads to the bottom of the cecum or ileocecal valve. Moreover, CT demonstrated swollen lymph nodes, and MRI revealed an epithelial tumor in the appendix. Thus, as we diagnosed LAMN or appendiceal cancer preoperatively in images, ileocecal resection with lymph node dissection was performed.

Imaging plays an important role in the diagnosis and management of mucinous appendiceal neoplasms. It might help diagnose asymptomatic neoplasms and allow detection of local invasion, impending or actual rupture, and metastatic spread of these tumors [6]. In this case, preoperative CT imaging showed no evidence of rupture or pseudomyxoma, as described earlier. In addition, fluid retention and appendiceal calcification were observed. On CT, only adenomas are identifiable as tumors confined to the appendix with no peri-appendiceal mucin. However, with continued mucin production and neoplastic expansion, mucinous neoplasms can develop focal mural expansions or a blistered appearance, and can lead to rupture due to mucin dissecting the wall [6]. In this case, MRI also suggested the presence of an epithelial tumor in the appendix. It revealed a high-intensity signal on DWI suggestive of adenocarcinoma. A previous report suggested that mucinous neoplasms appear as hyperintense tubular distension of the appendix on T2-weighted MRI images. Contrast-enhanced MRI may show smooth mucosal enhancement in simple lesions, whereas nodules and solid components are seen in adenomas, LAMNs, and adenocarcinomas. Peri-appendiceal enhancement may be seen when there are adhesions or extraluminal mucin, which raises the possibility of an underlying LAMN or adenocarcinoma. There is some evidence suggesting that DWI may be superior to CT in evaluating the extent of peritoneal disease [6,9].

In our case, we determined that appendiceal cancer could not be ruled out based on the preoperative DWI showing a high-intensity signal in the appendix. However, pathological results confirmed that it was LAMN. Thus, preoperatively, it is difficult to strictly distinguish LAMN, which can be cured by a simple appendectomy, from mucinous adenocarcinoma, which requires ileocecal resection with lymph node dissection.

We experienced five cases of LAMN at our hospital from 2010 to 2019 (Table 1). PMP was observed in one patient, three underwent emergency surgery, and ileocecal resection was performed on two patients, including the patient reported here. There was no case of lymph node metastasis. These results are compatible with what has been reported above.

**Table 1:** Patients characteristics of diagnosed LAMN in our hospital.

No.	Age	Sex	Preoperative Diagnosis	Tumor Size (mm)	Emergent Operation	Procedure	Ascites	No. of LN metastasis	No. of LN harvest
1	55	F	Appendiceal Mucinous Tumor	65 × 50	+	ICR	–	0	30
2	95	M	Appendiceal Abscess	68 × 51	+	ICR	–	0	7
3	81	F	Appendiceal Mucinous Tumor	86 × 30	–	CR	–	0	0
4	73	M	Appendiceal Mucinous Tumor	64 × 55	–	CR	–	0	0
5	67	F	PMP	55 × 29	+	CR	+	0	0

LN: lymph node, ICR: ileocecal resection, CR: partial cecum resection, PMP: pseudomyxoma peritoni.

## 4 Conclusion

Appendectomy is considered appropriate if the tumor margin can be secured. However, preoperative diagnosis is often inadequate, and ileocecal resection with lymph node dissection is necessary if appendiceal cancer is suspected. Further studies of preoperative imaging for appropriate differential diagnosis are necessary.

## Declaration of Competing Interest

The authors report no declarations of interest.

## Funding

Nothing to declare

## Ethical approval

This research was approved by the Ethics Committee and the Institutional Review Board at Kawakita General Hospital (No. 2020-0002) and was conducted according to the guidelines put forth in the Declaration of Helsinki.

## Consent

This case presentation was approved by the Local Ethics Committee of the Kawakita General Hospital (No. 2020-0002). The subjects were provided with the opportunity to opt-out. Therefore, no new consent for this study was required from the patient.

## Author contribution

YK made substantial contributions to study conception and design, acquisition of data, and analysis and interpretation of data. NU was involved in drafting the manuscript and revising it critically for important intellectual content. TK, SH, SA, TH, NT, AK and NT took part in the discussions about this study. All the authors read and approved the final manuscript.

## Registration of research studies

researchregistry6468 available at: https://www.researchregistry.com/browse-the-registry#home/

## Guarantor

Yusuke Kitagawa

## Provenance and peer review

Not commissioned, externally peer-reviewed
